# Antenatal and perinatal factors influencing neonatal blood pressure: a systematic review

**DOI:** 10.1038/s41372-021-01169-5

**Published:** 2021-08-07

**Authors:** Heike Rabe, Varsha Bhatt-Mehta, Stephen A. Bremner, Aisling Ahluwalia, Renske Mcfarlane, Simin Baygani, Beau Batton, Agnes Klein, Ebru Ergenekon, Luana Pesco Koplowitz, Eugene Dempsey, Dina Apele-Freimane, Hiroko Iwami, Janis M. Dionne, International Neonatal Consortium, Heike Rabe, Varsha Bhatt-Mehta, Stephen A. Bremner, Simin Baygani, Beau Batton, Agnes Klein, Ebru Ergenekon, Luana Pesco Koplowitz, Eugene Dempsey, Dina Apele-Freimane, Hiroko Iwami, Janis M. Dionne

**Affiliations:** 1 Brighton and Sussex Medical School, University of Sussex, Brighton, UK; 2 FDA Center for Drug Evaluation and Research, Virginia, MD USA; 3 Eli Lilly and Company, Indianapolis, IN USA; 4 Southern Illinois University School of Medicine, Springfield, IL USA; 5 Health Canada, Ottawa, ON Canada; 6 Gazi University, Ankara, Turkey; 7 DUCK FLATS Pharma LLC, New York, NY USA; 8 University College Cork, Cork, Ireland; 9 Pauls Stradins clinical university Hospital, Marupe, Latvia; 10 Osaka City General Hospital, Osaka, Japan; 11 British Columbia Children´s Hospital, Vancouver, BC Canada; 12 Critical Path Institute, Tucson, AZ USA

**Keywords:** Paediatrics, Medical research

## Abstract

**Objective:**

A comprehensive understanding of the factors contributing to perinatal blood pressure is vital to ensure optimal postnatal hemodynamic support. The objective of this study was to review existing literature on maternal and perinatal factors influencing blood pressure in neonates up to 3 months corrected age.

**Methods:**

A systematic search of published literature in OVID Medline, OVID Embase and the COCHRANE library identified publications relating to maternal factors affecting blood pressure of neonates up to corrected age of 3 months. Summary data were extracted and compared (PROSPERO CRD42018092886).

**Results:**

Of the 3683 non-duplicate publications identified, 44 were eligible for inclusion in this review. Topics elicited were sociodemographic factors, maternal health status, medications, smoking during pregnancy, and cord management at birth. Limited data were available for each factor. Results regarding the impact of these factors on neonatal blood pressure were inconsistent across studies.

**Conclusions:**

There is insufficient evidence to draw definitive conclusions regarding the impact of various maternal and perinatal factors on neonatal blood pressure. Future investigations of neonatal cardiovascular therapies should account for these factors in their study design. Similarly, studies on maternal diseases and perinatal interventions should include neonatal blood pressure as part of their primary or secondary analyses.

## Introduction

Blood pressure (BP) among newborn infants varies considerably in the immediate postnatal period [1–3]. Observed neonatal BP values have been associated with birthweight, gestational age at birth, and postnatal age [4, 5]. This variability in BP makes it challenging to know whether observed BP values are too high (hypertension), too low (hypotension), increasing too quickly, or increasing too slowly for a specific neonate during postnatal adaptation under specific clinical circumstances [4, 6, 7]. There is an additional need to address neonatal hypertension which is often underdiagnosed [2, 8, 9].

The impact of additional factors beyond gestational and postnatal age on neonatal BP values is unclear [1, 4,10–13]. These include maternal condition, perinatal clinical circumstances, and any additional, yet unclear, neonatal factors. A comprehensive understanding of these factors is vital to ensuring optimal provision of hemodynamic support for neonates in the immediate postnatal period.

Understanding the cause of hypotension allows for better therapeutic choices for postnatal neonatal hypotension treatment. For example, hypotension secondary to maternal anesthesia or analgesia may require reversal agents. Choice of fluid administration (in infants of insulin-dependent diabetic mothers) and/or choice of particular vasopressors such as dobutamine, milrinone, vasopressin, or dopamine for example may also be determined based on maternal factors. Septic shock may need more than one approach. Adrenal insufficiency may require early use of hydrocortisone.

The International Neonatal Consortium (INC) was formed in 2015 with the aim of engaging members of the global neonatal community to accelerate the advancement of safe and effective innovations in therapies for neonatal infants [14]. The Consortium comprises academic, clinical, industry, and nursing stakeholders as well as patient advocate groups and regulatory bodies, who are collaborating to collate existing evidence and combine it with their professional expertize to develop consensus-based guidance that can support future clinical trial methodologies.

This paper is the second in a series of articles being produced by the hemodynamic adaptation (HA) workgroup of INC. In the first instance the group worked on best practice recommendations for neonatal BP measurement methods [15]; the second instance is this article regarding maternal factors influencing neonatal BP during the first 3 months after birth; the third instance the group is working toward establishing observed “normal” BP ranges for neonatal infants of varying gestational ages based on a systematic review of available evidence, with the ultimate goal of establishing evidence-based approaches to assessment and management of neonatal circulation.

## Methods

This systematic literature review is developed based on a pre-specified protocol developed by the INC HA workgroup prior to initiation of the review. The protocol is registered on the PROSPERO database (ID CRD42018092886) [16].

### Eligibility criteria

Prospective and retrospective cohort studies, case series, and randomized controlled trials were all included during article selection. There was no limit on the publication year due to the importance of published evidence from early studies and therefore included literature from January 1946 to January 2017. Study populations included term and preterm neonates up to the corrected age of 3 months of all weights and in any health care context. Articles reporting neonatal BP as the main outcome, with analysis of maternal or perinatal factors were included. Papers with an absence of extractable data, invalid data analysis methods as determined by the statisticians in the HA workgroup and those published in a language not interpretable by any of the members of the HA workgroup were excluded.

### Search strategy

This systematic review was developed in accordance with the PRISMA guidelines (full checklist is available in *Supplementary data*) [17]. A systematic search of published literature was performed in OVID Medline, OVID Embase, the Cochrane Central Register of Controlled Trials (CENTRAL) and CINAHL. Papers were identified using the search terms (BP OR hypertension OR hypotension) AND (infant OR newborn OR neonate) AND infant [MeSH] AND (measurement OR normative) AND Humans [MeSH]). For finding articles related to cord management, additional search criteria Cord adj3 (clamp* or milk* or strip* or drain*) were used.

The initial search included papers relevant to three primary research aims developed by the HA workgroup to address neonatal HA and influencing factors during the first few hours and months after birth.

### Data extraction and synthesis

Content from the papers retrieved was organized by study details and each paper was assessed against the inclusion and exclusion criteria. Two independent reviewers screened the article titles and abstracts and applied the eligibility criteria in a blinded fashion to the full article once the article was selected based on abstract review. At this stage, all studies were assigned to the relevant sub-questions of the larger overarching aim of neonatal HA developed by the HA workgroup. Papers were eligible and selected for inclusion in this systematic review if they reported on maternal factors affecting neonatal BP.

All relevant summary statistics from the final selection of papers were extracted to Excel^®^ (Microsoft Office, Redmond, Washington, USA) regardless of statistical significance. Data comprised values for systolic blood pressure (SBP), diastolic blood pressure (DBP) and mean arterial pressure (MAP), recorded with mean and standard deviation (±SD) where possible, as well as direction, magnitude and significance of factor association, and description of results. Descriptive comparisons of such data are reported in this study. Due to the heterogeneity in the data reported, a meta-analysis was not performed.

### Assessment of risk of bias

The risk of bias was assessed by the two independent reviewers using criteria outlined in the Cochrane Handbook for Systematic Reviews of Interventions and an overall risk of bias score was given for each study using the Mixed Methods Appraisal Tool [18, 19]. Evidence selection bias was minimized by conducting a thorough literature search in five major databases to ensure all available data on the topic were included.

## Results

**Fig. 1:**
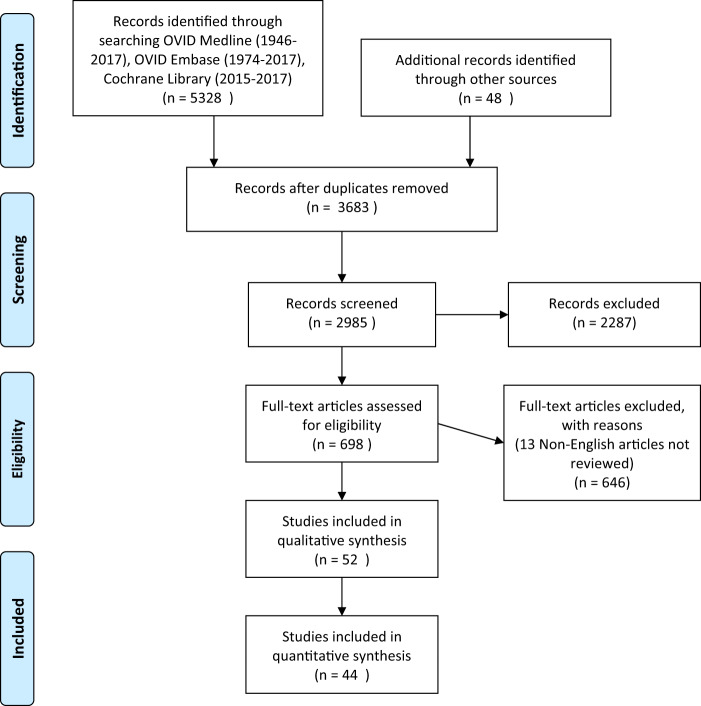
Selection of studies. PRISMA flow diagram.

The initial systematic search retrieved a total of 5376 papers, of which 3683 remained after removal of duplicate titles. A detailed evaluation of the elicited papers identified 52 studies that fit the inclusion criteria and a final 44 contained relevant data to be included in this review (Fig. 1). All 16 included papers related to maternal factors were prospective cohort studies published between 1976 and 2010 and descriptive characteristics are listed in Table 1. All studies included in this part of the review had a low risk for bias.

**Table 1:** Characteristics of included studies.

	Authors (Country)	Study type	*n*	Population (gestational age range)	Maternal factor	Indicator (number of subjects)	
1	Beratis, N.G., et al. [36] 1996 (Greece)	Prospective cohort	369	Term neonates born during a 6-month period in the Maternity Hospital of Patras (37–41)	Smoking in pregnancy	Non-smoking	(296)
						3–5 cigarettes/ day	(24)
						7–14 cigarettes/ day	(25)
						15+ cigarettes/ day	(24)
2	Geerts, C.C., et al. [37] 2007 (Netherlands)	Prospective cohort	456	Healthy term neonates in Leidsche Rijn (37–42)	Smoking in pregnancy	Non-exposed	(363)
						Exposed to others’ smoke	(63)
						Mother smoked	(30)
3	Czeszynska, M.B., et al. [25] 1999 (Poland)	Prospective cohort	89	Newborns born in the Department for Pathology of Pregnancy and Labor, Pomeranian Medical Academy in Szcezin, Poland during a 2-year period (23–41)	Blood pressure (Pre-eclampsia)	Normotensive (term)	(30)
						Pre-eclamptic (term)	(21)
						Normotensive (preterm)	(19)
						Pre-eclamptic (preterm)	(19)
4	Hegyi, T., et al. [26] 1994 (USA)	Prospective cohort	1105	Preterm neonates born/ transferred to neonatal intensive care units in the counties of Ocean, Monmouth, and Middlesex August 1984–June 1987 (Mean = 31.5, SD = 4.4)	Blood pressure (hypertension/ pre-eclampsia)	Normotensive	(244)
						Hypertensive	(47)
5	Hegyi, T., et al. [27] 1996 (USA)	Prospective cohort	991	Preterm neonates born/ transferred to neonatal intensive care units in the counties of Ocean, Monmouth, and Middlesex August 1984–June 1987 (Mean = 31.5, SD = 4.4)	Blood pressure (hypertension)	Normotensive	(183)
						Hypertensive mothers	(38)
6	Hernandez Arriaga, J.L., et al. [30] 1999 (Mexico)	Prospective cohort	72	Neonates 1500 and 2500 g born in Hospital General Regional de la Secretaría de Salud, Leon, Guanajuato, Mexico in March- August 1998 (NR)	Blood pressure (Pre-eclampsia)	Normative	[50]
						Pre-eclampsia	(22)
7	Kent, A.L., et al. [28] 2009 (Australia)	Prospective cohort	190	Neonates admitted to the neonatal intensive care unit (Mean = 35)	Blood Pressure (hypertension)	Term normotensive	(60)
						Term Hypertensive	(38)
						Preterm normotensive	(44)
						Preterm hypertensive	(14)
					Diabetes	Term non-diabetic	(60)
						Term Diabetic	(27)
						Preterm non-diabetic	(44)
						Preterm diabetic	(7)
8	Mausner, J.S., et al. [29] 1983 (USA)	Prospective cohort	391	Neonates enrolled at the Medical College of Pennsylvania and an affiliated hospital September 1977–March 1979 (NR)	Blood pressure (hypertension)	Healthy mothers	(38)
						Hypertensive	(60)
9	Gillman, M.W., et al. [20] 2004 (USA)	Prospective cohort	1059	Term neonates born in hospitals in Massachusetts (33.6–43.3)	Age	14–19 years	(NR)
					Blood Pressure	20–24	(NR)
						25–29	(NR)
						30–34	(NR)
						35–39	(NR)
						40–44	(NR)
						Maternal BP	(NR)
10	Sedaghat, N., et al. [22] 2008 (Australia)	Prospective cohort	406	Term neonates born between August 2003 and August 2005 (NR)	Age	Maternal age	(406)
11	Zinner, S.H., et al. [10] 1980 (USA)	Prospective cohort	837	Healthy Term neonates born in Boston City and the Women & Infants Hospitals of Rhode Island in Providence (37–41)	Age	Maternal age	(576)
					Race	White	(380)
						Black	(26)
12	Schachter, J., et al. [23] 1976 (USA)	Prospective cohort	247	Term neonates born in a large academic hospital (37–42)	Race	White	(136)
						Black	(111)
13	Schachter, J., et al. [24] 1982 (USA)	Prospective cohort	392	Healthy term neonates at normal birthweight (37–42)	Race	White	(197)
						Black	(142)
14	Sadoh, W.E., et al. [21] 2010 (Nigeria)	Prospective cohort	473	Term neonates born at the UBTH, Benin City and admitted to the postnatal ward (37–43)	Age	Maternal age	(473)
					Socioeconomic status	Low SEC	(NR)
						Middle SEC	(NR)
						High socioeconomic class	(NR)
					BMI	BMI < 25	(NR)
						BMI 25–30	(NR)
						BMI 30>	(NR)
15	Rantonen, T.H., et al. [31] 2002 (Finland)	Prospective cohort	40	Preterm neonates born at the Turku University Central Hospital (<33)	Magnesium sulfate treatment	Non-exposed	(12)
						Magnesium Sulfate treatment	(13)
					Ritodrine treatment	Non-exposed	(12)
						Ritodrine treatment	(15)
16	Yanowitz, T.B., et al. [38] 2002 (USA)	Prospective cohort	55	Preterm neonates born at Magee Women’s Hospital, Pittsburgh, Pennsylvania	Premature labor chorioamnionitis	On histology	(22
						control	(33)

*NR* not reported.

### Maternal sociodemographic factors

#### Maternal age

Gillman et al. studied 1059 full-term neonates to identify perinatal predictors of neonatal BP values, including maternal age. The authors reported a positive correlation between increasing maternal age and systolic BP of infants at 48 h of age suggesting that SBP among newborns was ~0.8 mm Hg higher for each increase of 5 years in maternal age. This correlation persisted even after controlling for potential confounding factors such as maternal high BP [20]. Zinner et al. found a positive correlation between maternal age and neonatal SBP and DBP in a subset (*n* = 576) of 837 maternal-infant pairs measured after uncomplicated vaginal or caesarean section deliveries [10]. The exact magnitude was not reported. A study by Sadoh, et al. of 473 mothers and infants found a lack of correlation between maternal age and neonatal SBP (*r* = 0.015, *p* = 0.374) [21]. However, every 10-year increase in maternal age was associated with an increase of 0.3 mmHg in neonatal SBP (neonatal SBP = 0.30 × age + 60.38, *R*^2^ = 98.6%). Pairwise interclass correlation coefficients were 0.196 for SBP and 0.157 for DBP (*p* < 0.001) [20]. A prospective cohort study of 406 term neonates reported no significant correlation between maternal age and neonatal SBP, DBP or MAP at either 24 or 48 h after birth [22]. With limited numbers of studies and conflicting results, definitive conclusions could not be made on the influence of maternal age on neonatal BP.

#### Maternal ethnicity/race

The effect of maternal ethnicity on neonatal BP is uncertain. Schachter et al. found higher DBP in term neonates of African-American mothers at 3 days after birth compared to white American infants (51.9 ± 6.7 mmHg versus 50.1 ± 6.6 mmHg; p = 0.047), but no significant difference in SBP was observed (76.4 ± 8.3 mmHg versus 75 ± 8.4 mmHg) [23]. In contrast, Zinner et al. reported no significant difference in SBP (74.1 ± 9.2 mmHg and 75.1 ± 11.2 mmHg respectively) or DBP (51.3 ± 9.0 mmHg and 51.3 ± 10.6 mmHg) in neonates born to white or African-American mothers [10]. Another prospective cohort study by Schachter et al. comparing 111 African-American with 136 white term newborn infants on day 3 after birth reported a marginally higher SBP for the African-American newborns (mean SBP 76.7 mmHg versus 74.3 mmHg; SD not reported; *p* = 0.04). However, when adjusted for number of feeds since birth, there was no longer a significant difference [24].

#### Maternal socioeconomic class

The mean SBP values of infants born to mothers from lower socioeconomic classes was reported to be significantly higher than that of infants of mothers from middle and high socioeconomic classes (70.8 ± 8.5 mmHg (low); 68.1 ± 8.2 mmHg (middle), 68.6 ± 8.3 mmHg (high) *p* = 0.022) in neonates in Nigeria [21]. Schachter et al. reported no effect of socioeconomic class on neonatal BP in infants at an academic hospital in the United States [23, 24].

### Maternal health status and diseases

#### Maternal body mass index (BMI)

Studies evaluating maternal BMI and neonatal BP in early life are scant. In a single study identified during this review, the mean SBP of infants of mothers with BMI < 30 was reported to be significantly lower than in infants whose mothers had BMI > 30 (*p* = 0.031) in a cohort of 473 Nigerian infants [21]. The exact SBP values for the newborn infants were not reported in the paper. This was also the case in some of the papers cited below and therefore exact BP values could not be reported.

#### Maternal blood pressure

In 2004, a study by Gillman et al. found a positive correlation between maternal BP and neonatal BP in 1059 maternal-infant pairs [20]. At 48 h after birth, there was an estimated 0.9 mmHg increase in neonatal SBP for every 10 mmHg rise in third trimester maternal SBP. Furthermore Czeszynska et al. reported that at 24 h after birth, term infants born to pre-eclamptic mothers had a significantly higher SBP (78.7 ± 10.9 versus SBP 74.4 ± 11.7 mmHg; *p* < 0.001) and DBP (44.4 ± 10.2 versus 41.2 ± 9.2 mmHg; *p* < 0.01) than those born to normotensive mothers (SBP 74.4 ± 11.7; DBP 41.2 ± 9.2 mmHg) [25]. DBP was higher in preterm neonates born to pre-eclamptic women (43.0 ± 9.2 mmHg versus 39.3 ± 8.8 mmHg; *p* < 0.001), but with no difference in SBP. Another study determined the effect of maternal BP in preterm infants (mean GA 31.5 weeks) and found that at 6 h after birth there were higher values for both SBP and DBP in preterm neonates born to mothers with hypertension compared to a control group of healthy mothers [26]. However, a subsequent paper regarding the same study sample reported no difference between preterm infants born to the normotensive and hypertensive mothers at 1 and 7 days of age. Fluctuations occurred over the days, resulting in no pattern of correlation [27].

In a 2009 study, Kent et al. found no correlation between BP in a cohort of 190 preterm and term infants born to normotensive compared with hypertensive mothers. At 14 days, there were no significant differences in SBP, DBP, or MBP in the term infant group. No difference in SBP, DBP, or MBP was found in the preterm infant group at 28 days after birth [28]. The study may have been underpowered for detecting differences. A 1983 study by Mausner et al. compared neonates born to a group of 201 normotensive mothers finding no differences between SBP and DBP in neonates between the two cohorts [29]. No significant difference in MBP in the first 3 days after birth was recorded between a small cohort of neonates born to pre-eclamptic mothers compared to normotensive mothers. Neonates of normotensive mothers had slightly higher MBP than pre-eclamptic mothers (48.1 mmHg and 47.5 mmHg, *p* value not provided), but this difference was not statistically significant and would not represent a clinically relevant difference [30]. With conflicting results in the identified studies, it is still uncertain whether maternal BP during and at the time of delivery has any effect on newborn BP.

#### Maternal diabetes

A study by Kent et al. showed no difference in SBP, DBP or MBP at 14 days post-delivery between term neonates born to mothers with diabetes compared with healthy mothers [28]. However, there were significantly higher readings for preterm neonates born to diabetic mothers at 28 days for SBP (67.4 mmHg versus 61.8 mmHg *p* < 0.001), DBP (37.7 mmHg versus 33.2 mmHg *p* < 0.02) and MAP (48.3 mmHg and 43.3 mmHg *p* < 0.01) none of which would be considered out of the normal ranges for this age group.

#### Maternal medications

Magnesium sulfate and ritodrine are tocolytic agents. Magnesium sulfate is also used in the treatment of severe pre-eclampsia and more recently as a neonatal neuroprotective agent in preterm deliveries. While generally safe, both of these agents can cause hypotension. Rantonen et al. [31] investigated the effect of maternal magnesium sulfate or ritodrine treatment on neonatal BP during the first 48 h after birth. They found no statistically significant difference between neonates exposed in-utero to magnesium sulfate (*n* = 13) or ritodrine (*n* = 15) and those not exposed to these agents (*n* = 12) although it was a small sample size [31].

The use of antenatal corticosteroids to prevent respiratory distress syndrome in preterm infants is common. Significantly higher mean BPs (up to 5 mmHg) have been reported in the first 24 h after birth in infants treated with a single course of antenatal corticosteroids. There is also a decreased need for inotropic support and fluid resuscitation during the first 24 h [32–34]. The effect of repeat courses of antenatal corticosteroids is less clear. In a randomized, blinded, placebo controlled clinical trial evaluating the effect of multiple courses of antenatal corticosteroids on neonatal BP and myocardial thickness, no difference was found between the placebo and repeat steroid groups [35].

#### Maternal smoking

A prospective cohort study by Beratis et al. demonstrated a positive correlation between the number of cigarettes smoked by mothers during pregnancy and BP in term infants within the first 72 h after birth [36]. The most marked observation was in infants born to mothers who smoked more than 15 cigarettes a day with significantly higher SBP (on average 12 mmHg higher at 72 h) and DBP (on average 8 mmHg higher at 72 h) at every time interval studied up to 24 months after birth. After 24 months, there was no significant difference in BP between infants of smoking and nonsmoking mothers [36]. Similarly, Geerts et al. [37] found that neonates of mothers who smoked during pregnancy had higher SBP (5.4 mmHg 95% CI: 1.2–9.7; *p* = 0.01) at 2 months of age compared with neonates who were not exposed to tobacco during pregnancy. No association was found between maternal smoking during pregnancy and neonatal DBP [37]. There was no difference in SBP or DBP between neonates who were born to non-smokers and to mothers who were exposed to secondary cigarette smoke. Further analysis for differences in gender showed that male neonates born to smoking mothers had 8.6 mm Hg higher SBP than those born to nonexposed mothers (*p* = 0.04).

#### Chorioamnionitis

There were only two studies reporting on the association between chorioamnionitis and neonatal BP.

A prospective observational cohort study by Been et al. [36] of 271 preterm infants born at ≤32 weeks gestation studied BP during the first 72 h after birth which was correlated with the use of antenatal steroids and histological evidence of chorioamnionitis. Infants whose mothers were diagnosed with chorioamnionitis had lower mean BPs especially during the first 12 h. In infants whose mothers received antenatal corticosteroids in the 7 days prior to delivery, the authors found an increase in mean arterial BP [36]. However, on multivariate analysis, maternal chorioamnionitis did not significantly affect neonatal BP. Antenatal corticosteroids, cord blood pH, and absence of maternal HELLP syndrome were associated with higher neonatal BP. Yanowitz et al. studied a cohort of 55 preterm infants <32 weeks gestation, including 22 with histologically confirmed maternal chorioamnionitis. They reported no significant differences in SBP at three (±1) hours after birth, but lower MBP and DBP for the group with chorioamnionitis (*p* < 0.05, exact data in mmHg not provided) [38].

#### Cord management

The standard approach to cord management at birth has been to clamp and cut the umbilical cord early (ECC) especially for infants born prematurely or those deemed to need resuscitation. However, in the past three decades, methods to enhance the transfer of placental blood to the baby have included Delayed Cord Clamping (DCC) or Umbilical Cord Milking (UCM) [39]. Our literature searches identified 24 papers in preterm infants (*n* = 1638 infants) [40–63] and four in term infants (*n* = 484 infants) [64–67] reporting on randomized trials comparing different cord management methods which are listed in Table 2. Of the preterm infant studies, five studies had no extractable data but these studies were included in the review as they had BP related comments within the text for comparative purposes [40, 42, 58, 60, 61]. Two papers were based on the same original cohort but the second paper reported additional data [55, 56].

**Table 2:** Study designs with comparison groups for placental transfusion methods and number of studies in preterm and term babies.

Preterm studies:	Term studies:
DCC versus ECC:14 papers	UCM versus ECC:2 papers
UCM versus ECC:8 papers	DCC long (300 s) versus DCC shorter (60 s):1 paper
UCM versus DCC:2 papers	UCM versus DCC:1 paper

*ECC* Early cord clamping, *DCC* Delayed cord clamping, *UCM* Umbilical cord milking, *s* seconds.

Study designs of comparison groups are listed in Table 3 (see online supplementary). All studies were at high risk for performance bias as placental transfusion cannot be blinded for the practitioners. Many of the studies were unclear for other aspects of risk of bias. Certainty of the evidence (CoE) using GRADE was mostly low, mainly due to imprecision and unclear risk of bias. The studies are listed in Table 2. Overall, the randomized controlled studies were difficult to compare as study designs were heterogeneous in terms of methods chosen and timing and degrees of the placental transfusion. In the preterm studies (14 studies, 850 infants), DCC timings were set from 30 to 90 s with a median of 30–45 s.

**Table 3:** Characteristics of randomized controlled trials on cord management.

Authors	*n*	Population and gestation	Intervention, time in seconds and positioning with regards to placenta level	Control group, time and positioning with regards to placenta level	Main blood pressure (mmHg) findings and measurement method
Preterm infants					
*Delayed cord clamping*					
Backes et al. 2016 [40]	40	Mother/single infant pairs, 22–27 weeks	DCC 30–45 s, baby held low	ECC 5–10 s	During first 24 h of life MBP was lower in ECC group than in DCC group (*p* < 0.05). DCC had higher MBP (mean difference of 4.13 mmHg, 95% CI 2.0–6.2, *p* < 0.01) (intra-arterial line). No extractable data
Baenziger O., et al. 2007 [41]	39	Mother/infant pairs, 24–32 weeks	DCC 60–90 s, baby held low	ECC < 20 s	MBP higher in experiment group compared to control group at 4 h but did not differ at 24 and 72 h. (MBP, NR)
Dipak et al. 2017 [42]	78	Mother/infant pairs, 27–31.6 weeks	DCC (with and without ergometrine), 60 s, baby held low	ECC < 10 s	MBP higher in DCC group. Mean difference between groups ECC and DCC (no ergometrine) was MBP at 12 h: 10.2(±2.3), *p* < 0.001 ECC and DCC (with ergometrine) 10.3 (±2.3) *p* < 0.001 (MBP, Non-invasive) No extractable data
Dong et al. 2007 [43]	90	Mother/infant pairs, <32 weeks	DCC 45 s, baby held low	ECC > 10 s	Higher MBP in DCC group MBP < 1 h: DCC 47(±6) ECC 42 (±8) *p* < 0.001
Gokmen et al. 2011 [44]	42	Mother/infant pairs, 24–31.6 weeks	DCC 30–45 s, not reported	ECC < 10 s	Initial MBP was higher in DCC group MBP < 1 h: DCC 42.8 (±6.5) ECC 39.4 (±8.9) *p* < 0.05
Hofmeyr et al. 1988 [45]	38	Mother/infant pairs, <35 weeks	DCC 60 s, not reported	ECC < 10 s	No statistically significant difference between groups (SBP, NR) No data in text.
Kugelman et al. 2007 [46]	65	Mother/infant pairs, <35 weeks	DCC 30–45 s, baby held low	ECC < 10 s	Initial MBP on admission to NICU in neonates <1500 g tended to be higher in DCC group, in the total cohort and in the section deliveries (SBP/ DBP/MBP, NR). MBP < 1 h neonates <1500 g, section: DCC 44 (±11) ECC 36 (±7) *p* = 0.05
Mercer et al. 2003 [47]	32	Mother-infant pairs <32 weeks, vaginal or c-section delivery	DCC 30–45 s, baby held low	ECC 5–10 s	Adjusting for gestational age, infants in the DCC group were three times more likely to have mean BP above 30 mmHg (Dinamap) MBP < 4 h: DCC 35 (±7) ECC 30 (±4.6) *p* = 0.017
Mercer et al. 2006 [48]	72	Mother/infant pairs, <32 weeks	DCC 30–45 s, baby held low	ECC 5–10 s	No significant difference MBP in first 4 h (data NR)
Mercer et al. 2016 [49]	211	Mother/infant pairs, 24–31.6 weeks	DCC 30–45 s, baby held low (UCM 1× before clamping or UCM 2–3x if could not do DCC)	ECC < 10 s	MBP: No significant difference between groups (Dinamap)
Nelle et al. 1998 [50]	19	Mother/infant pairs, <32 weeks	DCC, 30 s, baby held low;	ECC < 10 s,	DCC improves MBP (Dinamap)
Oh et al. 2011 [51]	33	Mother/infant pairs, 24–27 + 6/7 weeks	DCC 30–45 s, baby held low	ECC < 10 s	Hourly MBP ranged between 26 and 32 mm Hg during the first 12 h. No difference was observed between the two groups (no exact data) (intra-arterial catheter or Dinamap)
Popat et al. 2018 [52]	51	Infants aged <6 h and <30 weeks	DCC < 60 s, baby held low	ECC < 10 s	Infants with DCC had a higher diastolic blood pressure at 12–28 h of age (MBP/SBP/DBP, indwelling arterial catheter) DBP 24 h: DCC 30(±5) ECC 26 (±5) *p* < 0.05
Rabe et al. 2000 [53]	40	Mother/infant pairs <33 weeks	DCC, 45 s, baby held low	ECC, < 20 s	No significant differences between groups (SBP/DBP/MAP, NR)
*Umbilical cord milking*					
El-Naggar et al. 2016 [54]	73	Mother/infant pairs 24–30 + 6/7 weeks	UCM, 3×, baby held at placental level or below	ECC, < 10 s	No statistically significant difference between MBP UCM and ECC group at 4–6 and 10–12 h (ultrasound)
Hosono et al. 2008 [55]	40	Mother/infant pairs 24–28 weeks	UCM 2–3×, baby held at or below placenta level	ECC < 10 s	UCM was associated with higher blood pressure 34(±9) than controls 28(±8; *p* = 0.03. (SBP/DBP/MBP, arterial catheter) MBP < 1 h:UCM 34(±9) ECC 28(±8) *p* < 0.03 SBP < 1 h: UCM 45(±11) ECC 38 (±10) *p* < 0.05 DBP < 1 h: UCM 29(±8) ECC 23(±7) *p* < 0.04
Hosono et al. 2009 [56]	40	Same study as above but extended	UCM 2–3×, baby held at or below placenta level	ECC < 10 s	(SBP/DBP, arterial catheter) Graph only, showing *p* < 0.05 in favour of UCM for <1 h, 6 h, 12 h
Katheria, A.C., et al. 2014 [57]	60	Pregnant women <32 weeks gestation	UCM 3×, baby held low	ECC, NR	At time of first Echocardiogram (<6 h), UCM neonates had slightly higher DBP and MBP (MBP/SBP/DBP, Oscillometric) MBP 6 h: UCM 41(±9) ECC 36 (±9) *p* < 0.05 DBP 6 h: UCM 34 (±8) ECC 29 (±10) *p* < 0.001
Katheria et al. 2015 [58]	197	Mother/infant pairs <32 weeks, scheduled for sectio	UCM 4×, baby held low	DCC 45–60 s, baby held low	MBP was higher in UCM group for first 15 h of life (*p* < 0.02) (Ultrasound) Graph only
Kumar et al. 2015 [59]	125	Mother/infant pairs, 32–36 + 6/7 weeks	UCM after cutting, 3×	ECC < 10 s	MBP at 30 min higher in UCM group (NR)
March et al. 2013 [60]	75	Mother/infant pairs, 24–28 weeks	UCM 3×, baby at level of or below placenta	ECC < 10 s	No significant differences between groups (SBP/DBP, NR)
Rabe et al. 2011 [61]	58	Mother/infant pairs, 24–32 + 6/7 weeks	UCM 4×, baby held low	DCC 30 s, baby held low	No significant differences between groups (MBP, NR)
Ram-Mohan et al. 2018 [62]	60	Mother/infant pairs <37 weeks, infant requiring resuscitation at birth	UCM after cutting, 25 cm 4×	ECC NR	Babies in milking group had higher MBP at 6 h (*p* = 0.04) and 24 h of life. (MAP, multichannel monitor) MBP 6 h:UCM 50.36 (7.0) ECC 45.3 (7.5) *p* < 0.04
Song et al. 2018 [63]	66	Mother/infant pairs, 24–36 + 6/7 weeks	UCM 4×, baby held low	ECC < 10 s	No significant differences between groups. (MBP, NR)
*Term infants*					
Erickson-Owens et al. 2012 [64]	24	Mother/infant pairs, 37–41 + 6/7 weeks, C-sections	UCM 5×, at or below placenta	ECC < 10 s	No significant difference observed between groups (MBP, Dinamap)
Jaiswal et al. 2015 [65]	200	Mother/infant pairs, 36> weeks	DCC 60–90 s, baby at level of placenta	UCM, cut cord 3×, 25 cm, baby at level	No significant difference observed between groups MBP (oscillating NIBP (Schiller) in right arm using a size “0” cuff for term babies with bladder dimension of 6 cm)
Katheria et al, 2017. [66]	60	Infants >37 weeks needing attendance of a neonatal health care provider; infants with a fetal heart rate (HR) tracing showing minimal-absent variability, recurrent fetal HR decelerations, prolonged tachycardia or bradycardia, shoulder dystocia, fetal malpresentation, vacuum- or forceps-assisted vaginal delivery, and meconium-stained amniotic fluid	DCC 300 s	DCC 60 s	Mean BP values were significantly greater at 12 h of life in the infants in the 5 min DCC group (Dash 3000) MBP 12 h: DCC 300 s: 53 (±13) DCC 60 s: 47 (±7) *p* = 0.02
Upadhyay et al. 2013 [67]	200	Mother/infant pairs, >35 + 6/7 weeks	UCM, after cutting 3×, 25 cm, baby held at level of incision	ECC < 10 s	Study demonstrated relatively higher blood pressure (although within normal range) over an initial period of 48 h in milked group MBP < 1 h: UCM 51.6 (±11.3) ECC 48.4 (±10.7), *p* < 0.05. MBP 12 h: UCM 50.6 (±10.8) ECC 47.3 (±9.5), *p* < 0.05. MBP 48 h: UCM 50.3 (±11) ECC 46.2 (±9.2) *p* < 0.05.

*ECC* Early cord clamping, *DCC* Delayed cord clamping, *UCM* Umbilical cord milking, *NR* not reported, *MBP* Mean blood pressure, *SBP* Systolic blood pressure, *DBP* Diastolic blood pressure.

Similar variations were seen in UCM (10 studies, 788 infants), where the number of times milked varied between two to fourfold. Furthermore, eight studies milked before clamping/cutting the cord, and two cut the cord before milking the remaining cord stump, mostly due to the perceived need to resuscitate at birth.

The definition of immediate ECC ranged from 10 to 20 s. Only four studies (484 infants) reported BP data in term infants born after receiving placental blood and these were highly heterogeneous [64–67]. The gestational age at birth for preterm infants in the eligible studies varied widely and there was inconsistency in study design.

Fourteen studies compared DCC versus ECC. These studies are described in Table 2. Five studies found no difference in mean BP between groups while eight studies did report a difference (six of which reported statistically significant differences between groups in favor of DCC;), which resulted in less need for inotropes.

**Fig. 2:**
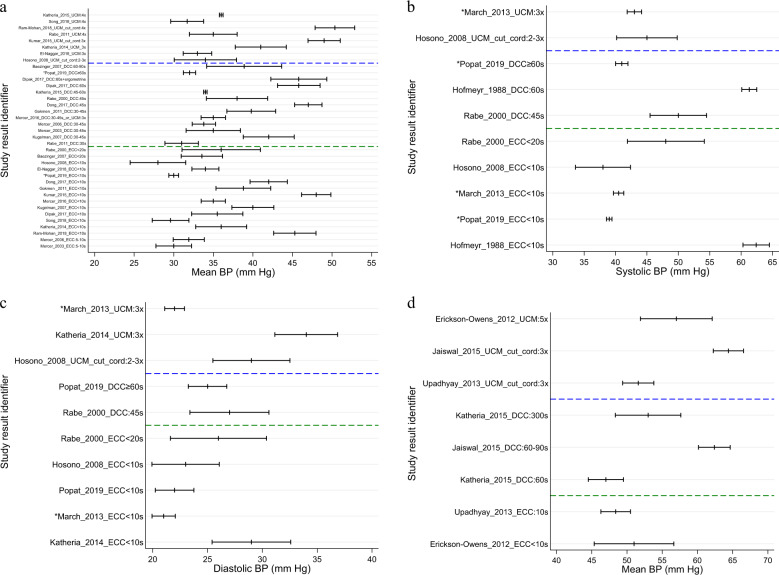
Blood pressure values in relation to cord management methods and infant maturity. **a** Mean blood pressure (mm Hg) and 95% confidence interval in studies of umbilical cord milking, delayed and early cord clamping in preterm infants. *Variance estimated as range/4. **b** Systolic blood pressure (mm Hg) and 95% confidence interval in studies of umbilical cord milking, delayed & early cord clamping in preterm infants. *Variance estimated as range/4. **c** Diastolic blood pressure (mm Hg) and 95% confidence interval in studies of umbilical cord milking, delayed & early cord clamping in preterm infants. *Variance estimated as range/4. **d** Mean blood pressure (mm Hg) and 95% confidence interval in studies of umbilical cord milking, delayed & early cord clamping in term babies.

Seven studies looked at UCM versus ECC. Three reported no difference between cohorts [54, 60, 66]. Four reported statistically significant difference with an average increase in mean BP by as much as 6 mmHg in infants after UCM as compared to ECC. Focusing on SBP, DBP and MBP in the first hour after birth, at 4 h, and at 24 h allows for some comparison of all studies in preterm and term infants with extractable data (Fig. 2a–d). The illustration demonstrates a trend toward higher values for DBP and SBP with longer cord clamping times or milking of the intact cord.

Two studies looked at UCM versus DCC. One reported no difference in neonatal BP. The other study reported a statistically significant difference in mean BP with higher values reported after UCM.

In term infants, two studies reported no differences in neonatal BP values between cohorts whilst the study comparing shorter DCC (60 s) versus longer DCC (300 s) reported long DCC favorable and 1 comparing UCM versus ECC showed a statistically significant difference in favor of UCM. Due to the high number of studies where the device used for BP measurements was not reported, it is not possible to link outcomes with type of BP device used.

## Discussion

Only 44 papers met inclusion criteria for this systematic review—a relatively low number given the breadth of the topic and range of years included. However, all of the included studies which covered the topics of maternal conditions and medications had a low bias risk, and yielded results from 7172 mother-infant pairs. This literature searches and incorporated studies provide interesting data on the impact of maternal socio-demographics, health status during pregnancy, maternal smoking, and antenatal medications on neonatal BP values [5, 20, 23, 27, 28, 33, 35, 36].

The included studies present mixed results regarding the impact of maternal factors on the BP in neonates. Maternal age is reported to be positively associated with neonatal BP in some studies [10, 20], but not others [23, 24]. Similarly, studies investigating associations between maternal social class and ethnicity and neonatal BP report mixed results [21, 23, 24]. Pregnancy related maternal diseases (e.g., maternal hypertension, diabetes or other medical conditions) appear to be associated with increased neonatal BP, but to a variable extent [26, 27].

It can be concluded that maternal age, the advancement of which was shown to correlate with an increase in neonatal BP in two out of four papers, has presented as the most important associated sociodemographic factor in determining BP at birth, in this review [10, 20, 21, 24]. There are inconsistencies among the studies reporting on associations between ethnicity and socioeconomic status of mothers on neonatal BP, leading to insufficient evidence to draw conclusions on whether or not these factors have a significant impact [21, 23]. In addition, multiple factors may be occurring in individual patients and studies could have difficulty in separating out the influences.

This review found wide variations in reported associations between maternal BP and neonatal BP. Clearly positive correlations were identified in some studies, although this is inconsistent throughout all papers and there is ambiguity between findings, sometimes even within the same study [20,25–30]. Discrepancies between the studies around age of neonates at the time of BP measurements poses challenges when comparing the outcomes, due to the rapidly evolving haemodynamic state of infants over the first weeks after birth [1, 2]. Although this review highlights maternal BP as a potential factor affecting neonatal BP without strong evidence in large scale studies, no definitive conclusions can be drawn from the available evidence and therefore, further research is required. Only one study reported on the effect of BMI on neonatal BP with a significant difference between neonates of mothers with BMI > 30 compared with BMI < 30 [20]. Maternal BMI is known to affect maternal and fetal outcomes, although the exact cause of this is not fully understood [68]. In particular, infants born to mothers with high or low BMI experience more adverse effects than those of healthy mothers and are more likely to require neonatal hospital admission [68]. It would be beneficial, therefore, to investigate further the link between maternal BMI and neonatal BP. Diabetes in mothers is known to result in poorer neonatal outcomes, including cardiac pathologies. Only one paper reported on maternal diabetes and found that it was not correlated with neonatal BP [28]. Further research could provide insightful evidence around this topic [68]. Overall conclusion cannot be drawn from these single studies about the impact of maternal BMI and diabetes.

No correlation was noted, either between maternal use of magnesium sulfate or ritodrine during pregnancy and neonatal BP in the single study in which this was reported [31]. The use of antenatal steroids has been associated with higher neonatal BP [5]. Two studies reported a positive correlation between smoking during pregnancy and a higher BP of neonates [36, 37].

Overall, 24 papers related to placental transfusion in preterm infants, either through DCC or UCM, reported either no effect or an increase in BP measurement during the first 72 h after birth. The recently updated Cochrane Review found a benefit of placental transfusion in reducing the need for inotrope treatment for preterm infants during the first week after birth [39]. Not all randomized controlled trials reported on BP as a primary or secondary outcome measure, which should be correctly measured and reported in future studies. Likewise, drug studies during pregnancy should report both on shorter as well as longer term neonatal outcomes including BP and should record the cord management methods used.

Strengths of this review which increase validity include strict adherence to the PRISMA statement and pre-registering the study protocol on PROSPERO. Limitations include the small number of papers investigating each maternal factor and exclusion of papers published in a language not spoken by any member of the Consortium team. The methodological quality of included studies was disparate, particularly with regards to study design, patient population, methods of data analysis, and data presented. Potential explanations include the years elapsed since publication of several of the papers, challenges with data extraction—particularly variation in both the age at which BP values were obtained and type of BP (systolic, diastolic, mean) measured across studies, limited information or adjustment for possible confounding variables, and study differences in the neonatal population investigated.

### Implications for current practice and future research

The lack of concrete conclusions drawn from the available literature reflects the limited data on the topic surrounding the association between maternal factors and neonatal BP. The findings from this systematic review are not strong enough to impact current practice or offer generalizable information.

Future investigations of neonatal cardiovascular therapies should include both, maternal and perinatal factors in their study design and analysis and have adequate sample size. Similarly, studies on maternal diseases and perinatal interventions should include neonatal BP as part of their primary or secondary analyses. Understanding the cause of neonatal hypotension will allow for more targeted therapeutic interventions in the treatment of postpartum neonatal hypotension providing immediate effective therapies while avoiding adverse effects from “trial and error” approaches that utilize polypharmacy and are currently prevalent in the clinical arena.

## Conclusion

The aim of this systematic review was to assess the available published data regarding the influence of maternal factors on neonatal BP values. Ambiguity in the current published literature means that there is insufficient evidence to draw definitive conclusions about the extent to which certain maternal factors correlate with neonatal BP. There are some indications that maternal age, maternal BP, maternal BMI and maternal smoking have an effect, but data were insufficient to draw definitive conclusions or recommendations. There is a need to consider influential maternal conditions and therapies in future studies in order for a more complete understanding of factors contributing to the hemodynamic status of neonates in the immediate postnatal period. This review in conjunction with additional studies through the INC will assist with the development of evidence-based standards for neonatal protocols for hemodynamics therapy studies or understanding of normal or abnormal conditions to define adverse events.

## Supplementary information

Risk of bias assessment for randomized controlled trials related to cord management using Mixed Methods Appraisal Tools

Consortium Members

PRISMA Checklist

## References

[1] Pejovic B, Peco-Antic A, Marinkovic-Eric J (2006). Blood pressure in non-critically ill preterm and full-term neonates. Pediatr Nephrol..

[2] Dionne JM, Flynn JT (2017). Management of severe hypertension in the newborn. Arch Dis Child..

[3] Hulman S, Edwards R, Chen YQ, Polansky M, Falkner B (1991). Blood pressure patterns in the first three days of life. J Perinat Neonatal Nurs 1991..

[4] Lalan S, Warady B (2015). Discrepancies in the normative neonatal blood pressure reference ranges. Blood Press Monit..

[5] Kent AL, Chaudhari T (2013). Determinants of Neonatal Blood Pressure. Curr Hypertens Rep..

[6] Shead SL (2015). Pathophysiology of the Cardiovascular System and Neonatal Hypotension.. Neonatal Network..

[7] Baik N, Urlesberger B, Schwaberger B, Avian A, Mileder L, Schmölzer GM (2017). Blood Pressure during the Immediate Neonatal Transition: Is the Mean Arterial Blood Pressure Relevant for the Cerebral Regional Oxygenation?. NEO..

[8] Sharma D, Farahbakhsh N, Shastri S, Sharma P (2017). Neonatal hypertension. J Matern-Fetal Neonatal Med..

[9] Kraut EJ, Boohaker LJ, Askenazi DJ, Fletcher J, Kent AL (2018). Neonatal Kidney Collaborative (NKC). Incidence of neonatal hypertension from a large multicenter study [Assessment of Worldwide Acute Kidney Injury Epidemiology in Neonates-AWAKEN].. Pediatr Res..

[10] Zinner SH, Lee YH, Rosner B, Oh W, Kass EH (1980). Factors affecting blood pressures in newborn infants. Hypertension..

[11] Zubrow AB, Hulman S, Kushner H, Falkner B (1995). Determinants of blood pressure in infants admitted to neonatal intensive care units: a prospective multicenter study. Philadelphia Neonatal Blood Pressure Study Group. J Perinatol..

[12] Launer LJ, Hofman A, Grobbee DE (1993). Relation between birth weight and blood pressure: longitudinal study of infants and children. BMJ..

[13] Kilian K (2003). Hypertension in neonates: causes and treatments. J Perinat Neonatal Nurs..

[14] Turner MA, Davis JM, McCune S, Bax R, Portman RJ, Hudson LD (2016). The International Neonatal Consortium: collaborating to advance regulatory science for neonates. Pediatr Res..

[15] Dionne JM, Bremner SA, Baygani SK, Batton B, Ergenekon E, Bhatt-Mehta V (2020). Method of Blood Pressure Measurement in Neonates and Infants: a Systematic Review and Analysis. J Pediatrics..

[16] PROSPERO. International prospective register of systematic reviews. What are the observed ranges of blood pressure by gestational ages, birth weight, and age after birth without treatment? A systematic review. [Internet]. [cited 2020 Mar 5]. https://www.crd.york.ac.uk/prospero/display_record.php?RecordID=92886

[17] Moher D, Liberati A, Tetzlaff J, Altman DG. Preferred Reporting Items for Systematic Reviews and Meta-Analyses: the PRISMA Statement. PLoS Med [Internet]. 2009 Jul [cited 2019 Aug 7];6. https://www.ncbi.nlm.nih.gov/pmc/articles/PMC2707599/PMC3090117 21603045

[18] Cochrane Handbook for Systematic Reviews of Interventions [Internet]. [cited 2020 Feb 11]. /handbook/current

[19] Pluye P, Robert E, Cargo M, Bartlett G, O’Cathain A, Griffiths F, et al. (2011). Proposal: a mixed methods appraisal tool for systematic mixed studies, reviews. MIXED METHODS APPRAISAL TOOL (MMAT) VERSION 2018. mixedmethodsappraisaltoolpublic2.

[20] Gillman MW, Rich-Edwards JW, Rifas-Shiman SL, Lieberman ES, Kleinman KP, Lipshultz SE (2004). Maternal age and other predictors of newborn blood pressure. J Pediatrics..

[21] Sadoh WE, Ibhanesehbor SE, Monguno AM, Gubler DJ (2010). Predictors of newborn systolic blood pressure. West Afr J Med..

[22] Sedaghat N, Ellwood D, Shadbolt B, Kecskes Z, Falk MC, Brussel T (2008). The effect of mode of delivery and anaesthesia on neonatal blood pressure. Aust NZ J Obstet Gynaecol..

[23] Schachter J, Kuller LH, Perfetti C (1982). Blood pressure during the first two years of life. Am J Epidemiol.

[24] Schachter J, Lachin J, Wimberly F (1976). Newborn Heart Rate and Blood Pressure: relation to Race and to Socioeconomic Class. Psychosom Med..

[25] Czeszynska M, Pankiewicz E, Hnatyszyn G, Konefal H, Girzejowska M (1999). Neonatal blood pressure in relation to pregnancy-induced hypertension in mothers. Prenat Neonatal Med..

[26] Hegyi T, Carbone MT, Anwar M, Ostfeld B, Hiatt M, Koons A (1994). Blood pressure ranges in premature infants. I. The first hours of life. J Pediatrics..

[27] Hegyi T, Anwar M, Carbone MT, Ostfeld B, Hiatt M, Koons A (1996). Blood Pressure Ranges in Premature Infants: II. The First Week of Life. Pediatrics..

[28] Kent AL, Shadbolt B, Hu E, Meskell S, Falk MC, Dahlstrom JE (2009). Do maternal- or pregnancy-associated disease states affect blood pressure in the early neonatal period?. Aust NZ J Obstet Gynaecol.

[29] Mausner JS, Hiner LB, Hediger ML, Gabrielson MO, Levison SP (1983). Blood pressure of infants of hypertensive mothers: a two-year follow-up. Int J Pediatr Nephrol..

[30] Hernández JL, Malacara JM, Horta Domínguez I, Ricavar FE, de Jesús Hernández Hernández J, Hernández Arriaga JL (1999). Tensión arterial en recién nacidos de 1.500 a 2.500 gramos: estudio comparativo entre hijos de madres con pre-eclampsia y madres sanas [Arterial tension in newborns weighing 1,500 to 2,500 g: a comparative study between children of mothers with pre-eclamp.]. Rev Española Pediatría 1999..

[31] Rantonen TH, Grönlund JU, Jalonen JO, Ekblad UU, Kääpä PO, Kero PO (2002). Comparison of the effects of antenatal magnesium sulphate and ritodrine exposure on circulatory adaptation in preterm infants. Clin Physiol Funct Imaging..

[32] Demarini S, Dollberg S, Hoath SB, Ho M, Donovan EF (1999). Effects of Antenatal Corticosteroids on Blood Pressure in Very Low Birth Weight Infants During the First 24 h of Life. J Perinatol..

[33] Moïse AA, Wearden ME, Kozinetz CA, Gest AL, Welty SE, Hansen TN (1995). Antenatal steroids are associated with less need for blood pressure support in extremely premature infants. Pediatrics..

[34] Been JV, Kornelisse RF, Rours IGIJG, Passos VL, De Krijger RR, Zimmermann LJI (2009). Early Postnatal Blood Pressure in Preterm Infants: effects of Chorioamnionitis and Timing of Antenatal Steroids. Pediatr Res..

[35] Mildenhall L, Battin M, Bevan C, Kuschel C, Harding JE (2009). Repeat Prenatal Corticosteroid Doses Do Not Alter Neonatal Blood Pressure or Myocardial Thickness: Randomized, Controlled Trial. Pediatrics..

[36] Beratis NG, Panagoulias D, Varvarigou A (1996). Increased blood pressure in neonates and infants whose mothers smoked during pregnancy. J Pediatrics..

[37] Geerts Caroline C, Grobbee Diederick E, van der Ent Cornelis K, de Jong Brita M, van der Zalm Marieke M, van Putte-Katier Nienke (2007). Tobacco Smoke Exposure of Pregnant Mothers and Blood Pressure in Their Newborns. Hypertension..

[38] Yanowitz TD, Ann Jordan J, Gilmour CH, Towbin R, Bowen A, Roberts JM (2002). Hemodynamic Disturbances in Premature Infants Born after Chorioamnionitis: association with Cord Blood Cytokine Concentrations. Pediatr Res..

[39] Rabe H, Gyte GM, Díaz‐Rossello JL, Duley L (2019). Effect of timing of umbilical cord clamping and other strategies to influence placental transfusion at preterm birth on maternal and infant outcomes. Cochrane Database Syst Rev [Internet]..

[40] Backes C, Huang H, Iams J, Bauer J, Giannone P (2016). Timing of umbilical cord clamping among infants born at 22 through 27 weeks’ gestation. J Perinatol.

[41] Baenziger O, Stolkin F, Keel M, Siebenthal K, von, Fauchere J-C, Kundu SD (2007). The Influence of the Timing of Cord Clamping on Postnatal Cerebral Oxygenation in Preterm Neonates: a Randomized, Controlled Trial. Pediatrics..

[42] Dipak NK, Nanavati RN, Kabra NK, Srinivasan A, Ananthan A (2017). Effect of delayed cord clamping on hematocrit, and thermal and hemodynamic stability in preterm neonates: a randomized controlled trial. Indian Pediatr..

[43] Dong X-Y, Sun X-F, Li M-M, Yu Z-B, Han S-P (2016). [Influence of delayed cord clamping on preterm infants with a gestational age of <32 weeks]. Zhongguo Dang Dai Er Ke Za Zhi.

[44] Gokmen Z, Ozkiraz S, Tarcan A, Kozanoglu I, Ozcimen E, Ozbek N (2011). Effects of delayed umbilical cord clamping on peripheral blood hematopoietic stem cells in premature neonates. J Perinat Med.

[45] Hofmeyr GJ, Bolton KD, Bowen DC, Govan JJ (1988). Periventricular/intraventricular haemorrhage and umbilical cord clamping. Find Hypothesis S Afr Med J..

[46] Kugelman A, Borenstein-Levin L, Riskin A, Chistyakov I, Ohel G, Gonen R (2007). Immediate versus Delayed Umbilical Cord Clamping in Premature Neonates Born < 35 Weeks: a Prospective, Randomized, Controlled Study. Am J Perinatol.

[47] Mercer JS, McGrath MM, Hensman A, Silver H, Oh W (2003). Immediate and Delayed Cord Clamping in Infants Born Between 24 and 32 Weeks: A Pilot Randomized Controlled Trial. J Perinatol..

[48] Mercer JS, Vohr BR, McGrath MM, Padbury JF, Wallach M, Oh W (2006). Delayed Cord Clamping in Very Preterm Infants Reduces the Incidence of Intraventricular Hemorrhage and Late-Onset Sepsis: A Randomized, Controlled Trial. Pediatrics..

[49] Mercer JS, Erickson-Owens DA, Vohr BR, Tucker RJ, Parker AB, Oh W (2016). Effects of Placental Transfusion on Neonatal and 18 Month Outcomes In Preterm Infants: a Randomized Controlled Trial. J Pediatr..

[50] Nelle M, Fisher S, Conze S, Beedgen B, Grischke EM, Linderkamp O (1998). Effects of Late Cord Clamping on Circulation in Prematures (vlbwi). Pediatr Res.

[51] Oh W, Fanaroff AA, Carlo WA, Donovan EF, McDonald SA, Poole WK (2011). Effects of Delayed Cord Clamping in Very Low Birth Weight Infants. J Perinatol..

[52] Popat H, Robledo KP, Sebastian L, Evans N, Gill A, Kluckow M (2018). Interobserver agreement and image quality of functional cardiac ultrasound measures used in a randomised trial of delayed cord clamping in preterm infants. Arch Dis Child - Fetal Neonatal Ed..

[53] Rabe H, Wacker A, Hülskamp G, Hörnig-Franz I, Schulze-Everding A, Harms E (2000). A randomised controlled trial of delayed cord clamping in very low birth weight preterm infants. Eur J Pediatr.

[54] El-Naggar W. The Effect of Umbilical Cord Milking on Hemodynamic Status of Preterm Infants: a Randomized Controlled Trial [Internet]. clinicaltrials.gov; 2017 Mar [cited 2021 Jun 3]. Report No.: NCT01487187. https://clinicaltrials.gov/ct2/show/NCT01487187

[55] Hosono S, Mugishima H, Fujita H, Hosono A, Minato M, Okada T (2008). Umbilical cord milking reduces the need for red cell transfusions and improves neonatal adaptation in infants born at less than 29 weeks’ gestation: a randomised controlled trial. Arch Dis Child - Fetal Neonatal Ed..

[56] Hosono S, Mugishima H, Fujita H, Hosono A, Okada T, Takahashi S (2009). Blood pressure and urine output during the first 120 h of life in infants born at less than 29 weeks’ gestation related to umbilical cord milking. Arch Dis Child - Fetal Neonatal Ed..

[57] Katheria AC, Leone TA, Woelkers D, Garey DM, Rich W, Finer NN (2014). The Effects of Umbilical Cord Milking on Hemodynamics and Neonatal Outcomes in Premature Neonates. J Pediatrics..

[58] Katheria AC, Truong G, Cousins L, Oshiro B, Finer NN (2015). Umbilical Cord Milking Versus Delayed Cord Clamping in Preterm Infants. Pediatrics..

[59] Kumar B, Upadhyay A, Gothwal S, Jaiswal V, Joshi P, Dubey K (2015). Umbilical cord milking and hematological parameters in moderate to late preterm neonates: a randomized controlled trial. Indian Pediatr..

[60] March M, Hacker M, Parson A, Modest A, de Veciana M (2013). The effects of umbilical cord milking in extremely preterm infants: a randomized controlled trial. J Perinatol..

[61] Rabe H, Jewison A, Fernandez Alvarez R, Crook D, Stilton D, Bradley R (2011). Milking Compared With Delayed Cord Clamping to Increase Placental Transfusion in Preterm Neonates: a Randomized Controlled Trial. Obstet Gynecol.

[62] Mohan GR, Shashidhar A, Chandrakala BS, Nesargi S, Rao PNS (2018). Umbilical cord milking in preterm neonates requiring resuscitation: a randomized controlled trial. Resuscitation..

[63] Song S-Y, Kim Y, Kang B-H, Yoo H-J, Lee M (2017). Safety of umbilical cord milking in very preterm neonates: a randomized controlled study. Obstet Gynecol Sci.

[64] Erickson-Owens DA, Mercer JS, Oh W (2012). Umbilical cord milking in term infants delivered by cesarean section: a randomized controlled trial. J Perinatol..

[65] Jaiswal P, Upadhyay A, Gothwal S, Singh D, Dubey K, Garg A (2015). Comparison of two types of intervention to enhance placental redistribution in term infants: randomized control trial. Eur J Pediatr.

[66] Katheria AC, Brown MK, Faksh A, Hassen KO, Rich W, Lazarus D (2017). Delayed Cord Clamping in Newborns Born at Term at Risk for Resuscitation: a Feasibility Randomized Clinical Trial. J Pediatrics..

[67] Upadhyay A, Gothwal S, Parihar R, Garg A, Gupta A, Chawla D (2013). Effect of umbilical cord milking in term and near term infants: randomized control trial. Am J Obstet Gynecol.

[68] Kalk P, Guthmann F, Krause K, Relle K, Godes M, Gossing G (2009). Impact of maternal body mass index on neonatal outcome. Eur J Med Res..

