# Supplementary material for: Antenatal and perinatal factors influencing neonatal blood pressure: a systematic review
Source: J Perinatol. 2021 Aug 7;41(9):2317–29. doi: 10.1038/s41372-021-01169-5 (PMC8440188; doi:10.1038/s41372-021-01169-5)
Supplement: Supplementary file 1 — Risk of bias assessment for randomized controlled trials related to cord management using Mixed Methods Appraisal Tools [file 41372_2021_1169_MOESM1_ESM.docx]

(Suggested Supplement)Table 4: Risk of bias assessment for randomised controlled trials related to cord management using

Mixed Methods Appraisal Tools ^18,19^. L= low risk, U= unclear risk, H= high risk

| **Included study** | **Random sequence**  **generation** | **Allocation**  **concealment** | **Blinding of participants and personnel** | **Blinding of outcome assessment** | **Incomplete outcome data** | **Selective reporting** | **Other bias** |
| --- | --- | --- | --- | --- | --- | --- | --- |
| ***Preterm infants*** |  | | | | | | |
| ***Delayed Cord Clamping*** |  | | | | | | |
| **Backes 2016 (40)** | L | L | H | L | L | U | L |
| **Baenziger 2007 (41)** | U | U | H | U | H | H | H |
| **Dipak 2017 (42)** | L | L | H | U | L | U | U |
| **Dong 2007 (43)** | U | U | H | U | L | U | U |
| **Gokmen 2011 (44)** | U | U | H | L | L | U | U |
| **Hofmeyr 1988 (45)** | U | U | H | U | L | L | U |
| **Kugelman 2007 (46)** | U | U | H | U | L | U | L |
| **Mercer 2003 (47)** | U | U | H | U | L | U | L |
| **Mercer 2006 (48)** | L | L | H | U | L | U | L |
| **Mercer 2016 (49)** | U | U | H | L | L | U | U |
| **Nelle 1998 (50)** | U | U | H | U | U | U | U |
| **Oh 2011 (51)** | U | U | H | U | U | U | L |
| **Popat 2018 (52)** | L | L | H | L | L | U | L |
| **Rabe 2000 (53)** | L | L | H | U | L | U | L |
| ***Umbilical Cord Milking*** |  | | | | | | |
| **El-Naggar 2016 (54)** | L | L | H | L | L | H | U |
| **Hosono 2008 (55)** | U | U | H | U | L | U | L |
| **Hosono 2009 (56)** | U | U | H | U | L | U | L |
| **Katheria, 2014 (57)** | L | L | H | U | L | U | L |
| **Katheria 2015 (58)** | L | L | H | L | H | H | U |
| **Kumar 2015 (59)** | L | L | H | U | L | U | L |
| **March 2013 (60)** | L | L | H | H | U | U | L |
| **Rabe 2011 (61)** | L | L | H | U | L | U | U |
| **Ram-Mohan 2018 (62)** | L | L | H | U | U | L | L |
| **Song 2018 (63)** | L | L | H | H | L | L | L |
| ***Term infants*** |  | | | | | | |
| **Erickson-Owens 2012 (64)** | U | L | H | L | L | L | U |
| **Jaiswal 2015 (65)** | L | L | H | L | L | H | L |
| **Katheria 2017 (66)** | L | L | H | L | L | L | U |
| **Upadhyay 2013 (67)** | L | L | H | U | L | L | U |
