# Supplementary material for: Antenatal and perinatal factors influencing neonatal blood pressure: a systematic review
Source: J Perinatol. 2021 Aug 7;41(9):2317–29. doi: 10.1038/s41372-021-01169-5 (PMC8440188; doi:10.1038/s41372-021-01169-5)
Supplement: Supplementary file 2 — Consortium Members [file 41372_2021_1169_MOESM2_ESM.docx]

**Supplemental Information**

**International Neonatal Consortium**

**List of Members**

Karel Allegaert^1^, Judy Aschner^2^, Gerri Baer^3^, Jeffrey Barrett^4^, Norman Barton^5^, Ralph Bax^6^, Simin Baygani^7^, Sandra Beauman^8^, Debbie Bezalel^9^, Christina Bucci-Rechtweg^10^, Laura Butte^4^, Eammon Connolly^11^, Michael Cronin^5^, Edress Darsey^12^, Jonathan Davis^13^, Jennifer Degl^14^, Thomas Diacovo^15^, Janis Dionne^16^, Deborah Discenza^17^, Wakako Eklund^18^, Laura Fabbri^19^, Tsuyoshi Fukuda^7^, Christine Gleason^20^, Wolfgang Göpel^21^, Rahul Gupta^22^, Tiffany Halo^23^, Linda Han^5^, Rachel Hauser^22^, Heidrun Hildebrand^23^, Steven Hirschfeld^24^, Carole Kenner^25^, Agnes Klein^26^, Satoshi Kusuda^27^, Tamorah Lewis^28^, Sam Maldonado^29^, An Massaro^3^, Susan McCune^3^, Thomas Miller^23^, Jeffrey Ming^30^, Yeruk Mulugeta^3^, Hidefumi Nakamura^31^, Robert 'Skip' Nelson^29^, Victoria Niklas^5^, Gary Noel^32^, Michael Padula^33^, Antonello Pileggi^34^, Aprile Pilon^35^, Ronald Portman^10^, Heike Rabe^36^, Holly Robinson^7^, Klaus Romero^4^, Suna Seo^3^, Mary Short^38^, Angeliki Siapkara^39^, Doug Silverstein^3^, Kanwaljit Singh^4^, Roger Soll^40^, Keira Sorrells^41^, Sarah Spieth^4^, Robin Steinhorn^42^, Linda Storari^19^, Shari Targum^3^, Charlie Thompson^12^, Mark Turner^43^, Norma Vivas^30^, Kelly Wade^33^, Robert Ward^44^, Lisa Wiltrout^3^

^1^University of Leuven, Belgium

^2^The Joseph M. Sanzari Children’s Hospital at Hackensack Meridian Health, USA

^3^Food and Drug Administration (FDA), USA

^4^Critical Path Institute, USA

^5^Takeda Pharmaceutical Company, Japan

^6^European Medicines Agency (EMA), Netherlands

^7^Eli Lilly and Company, USA

^8^University of New Mexico Health Sciences Center, USA

^9^Bliss, UK

^10^Novartis Pharmaceuticals, Switzerland

^11^Infant Bacterial Therapeutics, Sweden

^12^Pfizer, Inc., USA

^13^Tufts Medical Center, USA

^14^Speaking for Moms and Babies, Inc., USA

^15^University of Pittsburgh Medical Center, Children’s Hospital of Pittsburgh, USA

^16^British Columbia Children’s Hospital, Canada

^17^Preemieworld, USA

^18^National Association of Neonatal Nurses (NANN), USA

^19^Chiesi Pharmaceuticals, Italy

^20^University of Washington, USA

^21^University of Lübeck, Germany

^22^March of Dimes, USA

^23^Bayer, Germany

^24^Uniformed Services University of the Health Sciences, USA

^25^Council of International Neonatal Nurses (COINN), USA

^26^Health Canada, Canada

^27^Kyorin University, Japan

^28^University of Missouri Kansas City (Children’s Mercy Hospital), USA

^29^Johnson and Johnson, USA

^30^Sanofi Pharmaceuticals, France

^31^National Center for Child Health and Development, Tokyo

^32^Institute for Advanced Clinical Trials for Children (I-ACT), USA

^33^Children’s Hospital of Philadelphia, USA

^34^National Institutes of Health (NIH)/NICHD, USA

^35^Trove Therapeutics, USA

^36^Brighton & Sussex Medical School, UK

^37^Pharmaceuticals and Medical Devices Agency (PMDA), Japan

^38^Retired Industry/Neonatal Nurse, USA

^39^Medicines and Healthcare products Regulatory Agency (MHRA), UK

^40^Vermont Oxford Network, USA

^41^NICU Parent Network, USA

^42^Rady Children’s Hospital, USA

^43^University of Liverpool, UK

^44^University of Utah, USA
